# Lutein supplementation in retinitis pigmentosa: PC-based vision assessment in a randomized double-masked placebo-controlled clinical trial [NCT00029289]

**DOI:** 10.1186/1471-2415-6-23

**Published:** 2006-06-07

**Authors:** Hossein Bahrami, Michele Melia, Gislin Dagnelie

**Affiliations:** 1 The Wilmer Eye Institute, Department of Ophthalmology, School of Medicine, Johns Hopkins University, Baltimore, MD, USA; 2 550 North Broadway, Suite 612, Baltimore, MD 21205, USA

## Abstract

**Background:**

There is no generally accepted medical or surgical treatment to stop the progressive course of retinitis pigmentosa. Previous studies have suggested lutein as a potential treatment with positive effects on macular pigment density. The objective of this study was to examine the effect of lutein supplementation on preservation of visual function in patients with retinitis pigmentosa (RP)

**Methods:**

In a double-masked randomized placebo-controlled phase I/II clinical trial with a cross-over design, 34 adult patients with RP were randomized to two groups. One group, consisted of 16 participants, received lutein supplementation (10 mg/d for 12 wks followed by 30 mg/d) for the first 24 weeks and then placebo for the following 24 weeks, while the other group included 18 participants for whom placebo (24 weeks) was administered prior to lutein. Visual acuity, contrast sensitivity, and central visual field were measured at different illumination levels at baseline and every week using a PC-based test at home.

**Results:**

For visual acuity (VA) at normal illumination level, treatment with lutein reduced logMAR, i.e. improved VA, but this effect was not statistically significant. The changes in normal (100%), low (4%), and very low (0.1%) illumination log CS were not statistically significant (p-values: 0.34, 0.23, and 0.32, respectively). Lutein had a statistically significant effect on visual field (p-value: 0.038) and this effect increased in the model assuming a 6-week delay in effect of lutein. Comparing the development of vision measures against the natural loss expected to occur over the course of 48 weeks, most measures showed reduced decline, and these reductions were significant for normal illumination VA and CS.

**Conclusion:**

These results suggest that lutein supplementation improves visual field and also might improve visual acuity slightly, although these results should be interpreted cautiously. As a combined phase I and II clinical trial, this study demonstrated the efficacy and safety of lutein supplementation.

## Background

Retinitis pigmentosa (RP) is the name used to designate a heterogeneous group of inherited progressive retinal dystrophies, characterized by photoreceptor degeneration, which initially affects the rod photoreceptors and the peripheral retina and often leads to legal and eventually functional blindness. It is estimated that about 1/5000 to 1/4000 individuals have RP worldwide[1,2]. Since no generally accepted medical or surgical treatment can stop the course of the disease, several researchers have undertaken informal or formal studies with vitamins and other nutritional supplements in hopes of improving patients' functional vision, or at least slowing down the course of the disease. A randomized controlled clinical trial demonstrated that vitamin A supplementation (15,000 IU/day) reduces the loss of the remaining electroretinogram; [3] despite some critical reviews, [4-6] it raised the hope for effective supplement-based treatments that can slow disease progression. Others have reported a beneficial effect of docosahexaenoic acid (DHA) in retinitis pigmentosa [7,8].

Lutein, as a carotenoid assumed to play a preventive role in macular diseases, [9] has been cited as a potential therapeutic modality that can help in preserving the visual function of patients with RP. Dietary modification and supplementation with lutein increase the macular pigment density [10,11] and several studies have shown the protective effect of long-term high dietary intake of carotenoids like lutein and zeaxanthin in reducing the risk of age-related macular degeneration. [12-14] A few uncontrolled studies have shown the effect of lutein supplementation in increasing the macular pigment density and improvement in visual acuity and central visual field (CVF) diameter in normally-sighted individuals and in RP and AMD patients [15-17]. These findings are suggestive of a possible benefit of lutein in RP patients. To our knowledge, the present study is the first randomized placebo-controlled double-masked clinical trial of lutein supplementation in Retinitis Pigmentosa; we could find no reference to similar studies in a computerized search of the major medical literature databases.

### Design

The study was a double-masked randomized placebo-controlled clinical trial with a crossover design. As it investigated both safety and efficacy of the supplements, and compared the effects of two supplementation levels to those of placebo, it can be classified as a combined phase I/II clinical trial.

## Methods

### Setting

The study was carried out with approval from the Johns Hopkins University, School of Medicine Institutional Review Board (IRB) and Informed Consent was obtained from the participants in accord with Helsinki Declaration and following careful explanation of all study conditions and procedures. The study was conducted at the Lions Vision Center, Wilmer Eye Institute, Johns Hopkins University between May 2001 and November 2002.

### Study population

**Figure 1:**
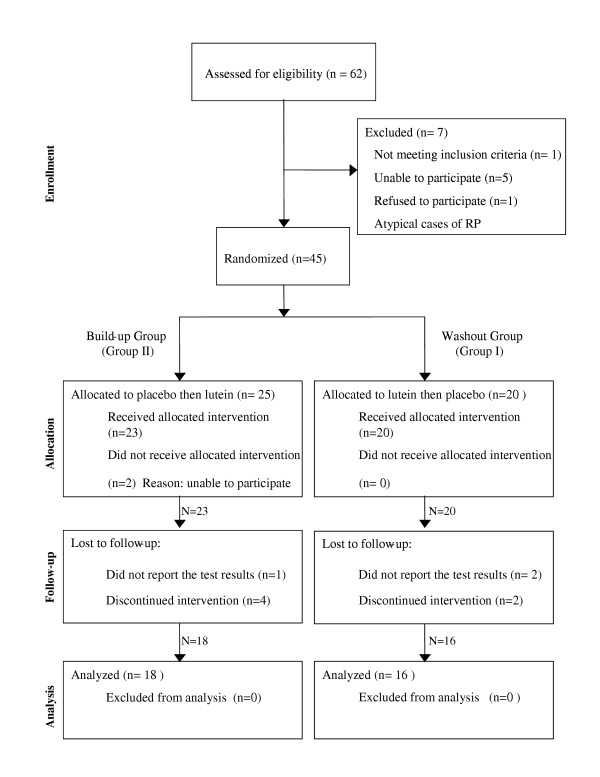
Flow diagram of study participants.

Participants were adult patients with diagnosed retinitis pigmentosa whose binocular central visual field was constricted to less than 30° from fixation in all directions. Patients were excluded from the study if at baseline: (1) their central vision was deteriorated beyond 20/100 visual acuity; or (2) they had abnormal liver function tests, defined by increase in liver aminotransferases, alkaline phosphatase, and serum bilirubin levels beyond the upper normal limit. Results for 34 participants completing the study, including 21 women and 13 men with a mean age 49.2 ± 9.0 years, are presented here. Figure 1 demonstrates the flow diagram of recruitment and group assignment process.

### Intervention

**Figure 2:**
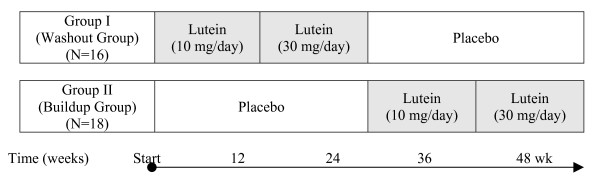
Allocation of lutein supplementation and placebo in two groups during The Trial Note: All participants received multi-vitamin supplement beginning 4 weeks before and continuing throughout the study.

The study population was divided into two groups and was followed for 48 weeks. Under a crossover design one group (washout group, group I), consisting of 16 participants, received lutein capsules during the first half of the study period (24 weeks) and indistinguishable placebo capsules during the second half, while the other group (buildup group, group II) included 18 participants who received placebo during the first half and lutein in the second half. Lutein supplementation in both groups started with 10 mg/day for 12 weeks followed by 30 mg/day for the following 12 weeks (Figure 2).

There have been suggestions in the literature that long-term supplementation of a single carotenoid may inhibit absorption of other carotenoids from the diet [18]. Therefore, all participants were offered multivitamin supplementation (Centrum Performance; 1 tablet/day), starting 4 weeks prior to the lutein/placebo supplementation.

### Randomization

Each participant received a unique subject code, consisting of a digit and a letter, e.g. 7a or 4b. The digit was drawn randomly by study group (odd for group I, even for group II); the letter represented the order of enrollment into the study group. All participants and study personnel were masked to the association of the odd/even assignment and supplementation order; this masking remained in effect until the end of the study. Randomization was stratified along five dimensions thought to have a possible correlation with lutein uptake and/or visual function in RP, i.e., gender, skin color (light vs. dark), iris color (blue, green/hazel, or brown), clinical evidence of cystoid macular edema (CME), and use of vitamin A supplements, so that near-equal numbers of participants with these characteristics were allocated to each group. A retina specialist examined prospective participants for cystoid macular edema, while the color of iris and skin were judged by the study coordinator. All supplement bottles were marked for subject ID and dispensing order, but otherwise indistinguishable; they were dispensed by the principal investigator.

### Outcome measures

Our specific objectives were to evaluate the effect of lutein on visual acuity, contrast sensitivity and central visual field, in comparison with placebo. The primary outcome measure for the study was ETDRS visual acuity recorded in the laboratory, supplemented with a number of secondary lab-based visual function measures. Outcomes recorded in the lab has been reported elsewhere [19]. Here we report on an alternate set of secondary vision measures collected weekly through personal computers. All measures were recorded in one eye only; this was usually the better eye, but in some participants, whose better eye had VA better than 20/20 in the lab (logMAR <0), the worse eye was selected as the study eye. Visual acuity (VA), contrast sensitivity (CS) and central visual field radius (CVF) were recorded at normal screen intensity, and using 4% (low; using NoIR U23 wrap-around shields) and 0.1% (very low; using welders' goggles with Schott #6 glass and Kodak Wratten ND1 filters) illumination. Visual acuity and contrast sensitivity were recorded as the logarithm of the minimum angle of resolution (logMAR) and the logarithm of contrast sensitivity (log CS), respectively. The visual field radius was measured along meridians at 15 degree (normal illumination; from 0 to 345 degrees) and 30-degree (low and very low illumination) intervals, and the mean radius was converted to the logarithm of the retinal area (in mm^2^) [20].

Participants installed and ran the test software under Windows; a secure file transfer protocol was used to upload the results to a server at our center. Validity and reliability of these tests was established independently [21]. The overall correlation between PC- and lab- measured VA and CS in normal illumination was 0.92 and 0.95, respectively (unpublished data). All participants performed the visual function tests weekly on their home or work PCs, keeping day and hour as constant as possible. Five participants who did not have access to a computer performed the same weekly tests on a PC in our lab. During the initial clinic visit, all subjects received careful instruction regarding the test, including a detailed manual. Also, on-screen reminders were designed before each test to help the subjects with correct setup conditions. To standardize test conditions as much as possible, all subjects were given 150 and 25 cm distance gages, to be attached to the PC monitor (25 cm for visual field, 150 cm for acuity and contrast sensitivity tests), a black eye patch to cover the non-tested eye, NoIR U23 filters and welders goggles to adjust the level of illumination, and a disposable camera, so the proper test setup and execution could be verified.

During each trial a test letter was shown in the center of the screen for 3 seconds or until the subject right-clicked the mouse to shorten the trial, and the subject then had to pick a match among 10 letters (C,D,H,K,N,O,R,S,V,Z). The visual field tests were based on the assumption that all patients had a central island, and the test algorithm flashed a dot along one of 24 axes (15° increments; 12 axes, i.e., 30° increments, at the two lower intensities), straddling the putative scotoma border; these tests are akin to Goldmann visual fields with a static (flashing) rather than kinetic (moving) test dot. All tests used a Bayesian adaptive algorithm [22] to find the threshold in a minimum number of trials: 24 for full intensity VA, 16 for all other VA and CS tests and for each CVF axis.

### Sample size and statistical analysis

The sample size calculation for this study was based on results from a pilot study of lutein supplementation, in which high contrast VA was the primary outcome[16]. The pilot study showed a mean effect of 7.0 letters and a mean standard deviation (SD) across subjects of 6.7 letters. With α = 0.05 and β = 0.20, and assuming a similar SD across subjects, we determined that a group size of 14 would be needed to detect an effect of 7.0 letters, or a group size of 26 to detect 5.25 letters (this reduced difference assumes a 25% placebo effect) [23]. These calculations did not take into account the increased power derived from multiple measurements. Since this was a phase I/II clinical trial, a sample size of 30–50 was considered prudent for this study. Analysis was performed based on intention-to-treat and using a multiple linear regression model with Generalized Estimating Equations (GEE) [24]. We also repeated the analysis using random effects and population-averaged linear mixed models. The two latter models are slightly different from GEE in the method of handling correlation in the data and, as expected, the analysis using these models did not demonstrate any significant differences, indicating the robustness of our models to the method of handling correlation. In these models, logMAR, log CS, and log retinal area were used as outcome variables and treatment (lutein vs. placebo), period (first vs. second 24-weeks of the study), time (as number of weeks within each period), age, the baseline value of each measure, vitamin A use (yes vs. no), iris color (in three categories; light/dark blue, green/hazel, and light/dark brown) and the interaction terms of treatment*time, period*treatment, and period*time were used as predictor covariates. Since we were interested in treatment effects and visual function test changes across the follow-up period, we forced time and treatment to the model even if they were found not to provide significant contributions. Other covariates were removed from the model in a backward stepwise elimination if they were found not to be significant. The most parsimonious models including significant and forced covariates are reported here. Baseline characteristics were checked for any imbalances. There was no significant difference in baseline characteristics of the subjects who withdrew did not complete the study and study participants. Since it was hypothesized that lutein effects might be delayed by about one visit to our center (6 weeks), a sensitivity analysis was done for each visual function measure to test this hypothesis.

## Results

### Baseline data

Data are reported here for the 34 participants who submitted a sufficient number of PC-based test data sets to allow reliable analysis of both study phases (at least 8 measures per study phase). Mean and standard deviation for the duration of PC-based data collection were 46.7 ± 13.7 weeks in the washout group, and 45.3 ± 8.9 weeks in the buildup group. Table 1 summarizes the baseline data for the two groups. None of the baseline data, except age (by which the participants were not stratified) and log CS at 4% illumination showed a considerable imbalance between two groups. Regarding the imbalance in age distribution, we included age in all GEE models and as explained below, it did not have a significant effect in any model, except in the model for contrast sensitivity at low (4%) illumination level.

**Table 1:** Baseline Values and Frequencies by Treatment Group

		Group I	Group II
Variable*		(Wash-out Group) (N = 16)	(Build-up Group) (N = 18)

Gender (Female to Male)		11/5	10/8

Age		52.4 ± 1.7	46.4 ± 2.3

Vitamin A Consumption		6	5

Iris color			
	Dark Brown/Light Brown	6	5
	Green/Hazel	6	6
	Dark Blue/Light Blue	4	7

Skin Color^†^			
	Pale	2	3
	Pale	10	14
	Light Brown	0	0
	Dark Brown	3	2

Cystoid Macular Edema		5	7

Visual Acuity (Log MAR)			
	Normal Illumination	0.275 ± 0.062	0.280 ± 0.050
	4% Illumination	0.497 ± 0.065	0.591 ± 0.072
	0.1% Illumination	0.902 ± 0.062	0.891 ± 0.065

Contrast Sensitivity (Log CS)			
	Normal Illumination	1.471 ± 0.105	1.346 ± 0.114
	4% Illumination	1.184 ± 0.090	0.928 ± 0.118
	0.1% Illumination	0.708 ± 0.102	0.658 ± 0.091

Log Retinal Area (square mm)		0.778 ± 0.336	0.970 ± 0.340

* Continuous variables are presented as mean ± standard error, while categorical and binomial variables are presented as absolute frequency.

† Skin color was defined based on the phenotype of the participant. Pale^+ ^refers to the very light skin color that burns rapidly in exposure to the sun.

### Lutein effects

The most significant predictors of visual function at all three levels of illumination were the baseline values (p value <0.001 in all models).

### Visual acuity

Table 2 summarizes the final GEE models for logMAR (visual acuity) at three levels of illumination, i.e. normal, low (4%), and very low (0.1%). According to the final model for visual acuity (log MAR) at normal illumination (table 2), treatment with lutein slightly reduced logMAR, i.e. slightly improved the VA, of the participants, but not to a statistically significant degree.

**Table 2:** Changes in Visual Acuity (LogMAR) at Different Levels of Illumination*

Level of Illumination	Normal (100%)		Low (4%)		Very Low (0.1%)	
	
Covariates^§^	Coefficient¶ (95% CI)	P value	Coefficient (95% CI)	P value	Coefficient (95% CI)	P value
Treatment^†^	-0.0002 (-0.0131,0.0128)	0.981	0.0054 (-0.0137,0.0246)	0.578	-0.0762 (-0.139, -0.0131)	0.018
Period	-^‡^	-	-0.0448 (-0.083,-0.0066)	0.022	-0.0654 (-0.134, -0.0132)	0.061
Time^† ^(number of weeks)	-0.0010 (-0.0026, 0.0007)	0.250	-0.0026 (-0.0048,-0.0003)	0.025	-0.0014 (-0.0027,-0.0001)	0.037
Age	-	-	-	-	-	-
Vitamin A Use	-	-	-0.0701 (-0.1357,-0.0046)	0.036	-	-
Iris color	-	-	-	-	-	-
Period × Treatment	-	-	-	-	0.1452 (0.0339, 0.2565)	0.011
Treatment × Time	-	-	-	-	-	-
Period × Time	-	-	0.0023 (0.0001, 0.0046)	0.044	-	-
Baseline LogMAR	0.9783 (0.8933, 1.0634)	<0.001	1.0086 (0.8909, 1.1268)	<0.001	0.9290 (0.8416,1.0164)	<0.001
Intercept	0.108 (-0.187, 0.402)	0.474	0.0265 (-0.0386, 0.0916)	0.424	0.0949 (0.0088, 0.181)	0.031

* Based on the GEE linear regression model

† Covariates forced to the model.

‡ Covariate removed due to non-significant contribution to the model (p value > 0.05).

§Description of covariates:

• Period: 0 in the first 24 weeks of the study, 1 in the second 24 weeks

• Treatment: 0 for placebo, 1 for lutein supplementation

• Time: Elapsed time within each 24-week period of the study (in weeks)

• Age: Age of the patients at baseline (in years)

• Vitamin A Use: 0 for participants not using, 1 for those using vitamin A supplements

• Iris color: Categorical variable: 1. Light/Dark Blue 2. Green/Hazel 3. Light/Dark Brown

¶The coefficient indicates the change in LogMAR units per unit change in the covariate.

At the low (4%) illumination level, participants ingesting vitamin A supplements tended to have better VA (p value: 0.04), independently of other covariates. The interaction between period and time was significant (p value: 0.04); therefore, it is not possible to interpret the effects of period and time directly from the model. Stratified analysis, using two models for the two periods, showed a slight non-significant reduction in logMAR of the participants who received lutein in first half of the study (p value: 0.57), while those who received lutein in second half of the study had a slight non-significant increase in logMAR (p value: 0.42). These results should be interpreted cautiously, since by stratification of data on period we lose some power of our crossover design.

In the model for VA at very low illumination level (0.1%), the interaction between period and treatment was significant (p value: 0.011), suggesting that the effect of treatment depends on period. If we insert the coefficients for period, treatment, and period*treatment into the model, then in the washout group this outcome measure was slightly better (lower) in the first than in the second half (-0.076 and -0.065, respectively). In the buildup group, the difference between average logMAR in the first and the second half of the study was very small (0.004).

### Contrast sensitivity

Table 3 summarizes the final GEE models for log CS (contrast sensitivity) at three levels of illumination, i.e. normal, low (4%), and very low (0.1%). The model for log CS at normal illumination shows a non-significant increase in log CS, i.e. improvement in contrast sensitivity, across time (p value: 0.31) after adjustment for period and type of treatment. Also participants on treatment with lutein showed a non-significant trend towards higher CS (p value: 0.34). But period did have a significant effect on log CS at normal illumination, suggesting that, controlled for type of treatment, log CS is higher during the second phase of the study than during the first phase (p value: 0.035). Stratified analysis of normal illumination log CS values showed a non-significant increase during both periods.

**Table 3:** Changes in Contrast Sensitivity (Log CS) at Different Levels of Illumination Ω

Level of Illumination	Normal (100%)		Low (4%)		Very Low (0.1%)	
	
Covariates^§^	Coefficient¶ (95% CI)	P value	Coefficient (95% CI)	P value	Coefficient (95% CI)	P value
Treatment^†^	0.0154 (-0.0164,0.0471)	0.342	0.0408 (-0.0273,0.1089)	0.241	-0.0184 (-0.0545, 0.0176)	0.316
Period	0.0351 (0.0025,0.0677)	0.035	0.0014 (-0.0853,0.0880)	0.975	-^‡^	-
Time^† ^(number of weeks)	0.0008 (-0.0008, 0.0025)	0.313	0.0001 (-0.0034, 0.0037)	0.934	-0.0009 (-0.0024,0.0006)	0.237
Age	-^‡^	-	-0.0049 (-0.0061,-0.0038)	<0.001	-	-
Vitamin A Use	-	-	-	-	-	-
Iris color	-	-	-	-	-	-
Period × Treatment	-	-	-	-	-	-
Treatment × Time	-	-	-	-	-	-
Period × Time	-	-	-0.0028 (-0.0055,-0.0001)	0.042	-	-
Baseline Log CS	0.9865 (0.9289, 1.0440)	<0.001	0.9451 (0.8496, 1.0406)	<0.001	0.9721 (0.9003,1.0440)	<0.001
Intercept	0.0133 (-0.0705, 0.0971)	0.755	0.2781 (0.1374,0.4188)	<0.001	0.459 (-0.0119, 0.1037)	0.119

Ω Variables and model criteria: See Table 2.

¶ The coefficient indicates the change in Log CS units per unit change in the covariate.

The low (4%) illumination log CS values were higher when participants were receiving lutein supplementation, which means an improvement in CS, but this change was not statistically significant (p value: 0.24). The model for CS at low (4%) illumination is the only model in which age had a significant effect, i.e. lower CS in older participants. Since there was a significant interaction between period and time in this model, the effect of these two variables could not be estimated directly from the coefficients, but this interaction suggests that low illumination log CS was declining in the second half of the study only. The model for CS at very low (0.1%) illumination was developed using the data from 32 cases, since two of the participants in the buildup group could not see the stimulus at this illumination level. No significant effects were found, other than the baseline value.

### Central visual field

In a similar model for log retinal area, participants did not have a significant change. The mean log retinal area when the participants were on lutein was 0.018 higher (95% CI: 0.001, 0.036, p value: 0.038) than when they received placebo, but the visual field deteriorated throughout the study (change per week: -0.2%, 95% CI: -0.003, -0.001, p value< 0.001). This reduction is consistent with about 11.4% decrease in log retinal area per year. Also participants who were taking vitamin A had a significantly larger visual field (difference in average log retinal area: 0.094; 95% CI: 0.008, 0.179, p-value: 0.032), but this difference did not change over the course of the study.

**Figure 3:**
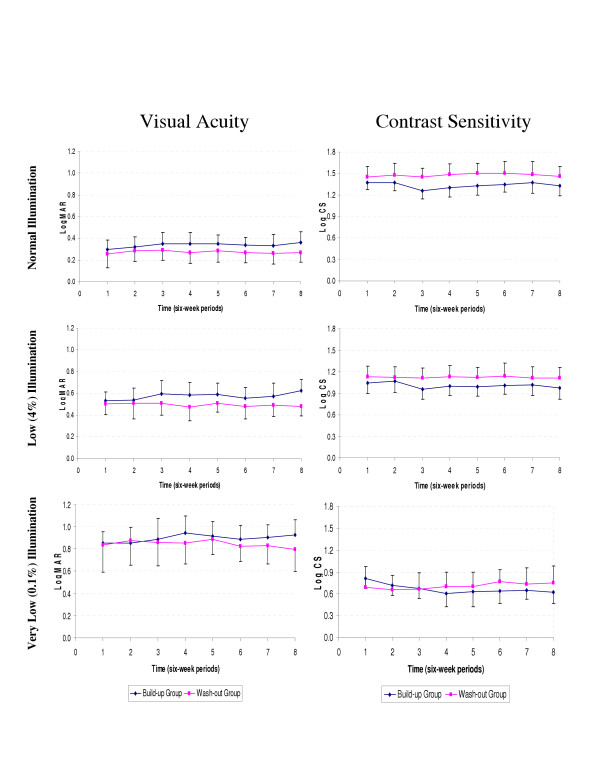
Changes of mean LogMAR and mean log CS at different illumination levels in Washout and Buildup groups. Note: Bars indicate 1.96*standard error

**Figure 4:**
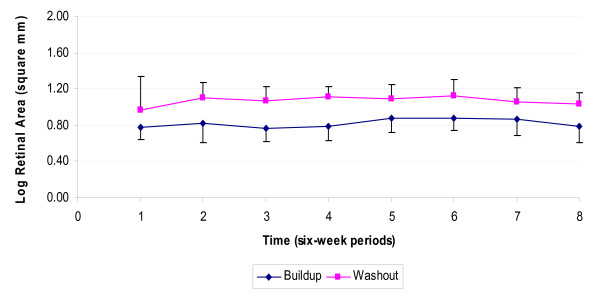
Changes of the visual field (mean log retinal area) at normal illumination level in Washout and Buildup groups. Note: Bars indicate 1.96*standard error

The overall changes in mean logMAR, mean log CS, and VF over time are illustrated in Figures 3 and 4. Mean values were averaged over six-week intervals; this might mask any within-interval changes, but this is not likely: The shallow slopes of the graphs are consistent with the small coefficients in our GEE models.

### Further analyses

Several sensitivity analyses were performed to verify the results and test the assumptions used in the analysis. Using a random effects model instead of population-average model and imputing missing values did not change the results of the study.

As another ancillary analysis we checked for a possible delayed effect of lutein supplementation, under the hypothesis that the effect of lutein may only appear after six or twelve weeks. Using a model with 6 weeks delay, the effect of lutein on VA at normal and low (4%) illumination levels did not show any qualitative changes compared to the model without delayed effect assumption. For the remaining measures some changes were found, however: The significant period effect observed for VA at very low (0.1%) illumination level was removed, while the treatment effect lost significance. Similarly, the period effect seen in the model for CS at normal illumination disappeared in the delayed effect model. Furthermore, the interaction between period and time for CS at low (4%) illumination lost significance. In this model, the average log CS at normal illumination was slightly (0.017 unit, 95% CI -0.014, 0.049, p value: 0.30) higher when participants were on lutein than when they were receiving placebo.

In the model assuming a 6-week delay effect of lutein on VF, lutein had a highly significant effect in preserving VF and the mean log retinal area when participants were on lutein was 0.029 (95% CI: 0.012, 0.047, p-value:0.001) higher than when they were on placebo. As expected, this change in analysis did not affect the rate of CVF loss, nor the observed constant difference between vitamin A users and non-vitamin A users. Table 4 summarizes the regression models for log retinal area without and with the 6-week delay assumption.

**Table 4:** Visual field regression model. Ω

Model	Without 6-Week Delay Assumption		With 6-Week Delay Assumption	
	
Covariates^§^	Coefficient¶ (95% CI)	P value	Coefficient (95% CI)	P value
Treatment^†^	0.0183 (0.0010,0.0355)	0.038	0.0292 (0.0118,0.0466)	0.001
Period	-^‡^	-	-	-
Time^† ^(number of weeks)	-0.0022 (-0.0034,-0.0010)	<0.001	-0.0023 (-0.0034,-0.0011)	<0.001
Age	-	-	-	-
Vitamin A Use	0.0937 (0.0081, 0.1793)	0.032	0.0941 (0.0080, 0.1803)	0.032
Iris color	-	-	-	-
Period × Treatment	-	-	-	-
Treatment × Time	-	-	-	-
Period × Time	-	-	-	-
Baseline Log Retinal Area	0.8965 (0.8309, 0.9621)	<0.001	0.8957 (0.8297, 0.9617)	<0.001
Intercept	0.1325 (0.0583, 0.2067)	<0.001	0.1299 (0.0554,0.2043)	0.001

Ω For description of variables and coefficients: See Table 2.

¶ The coefficient indicates the change in log (visual field area) per unit change in the covariate.

The other additional analysis performed was a comparison between changes in visual function under treatment and the natural course of retinitis pigmentosa, considering that this natural course entails a gradual deterioration of visual function. The rate of this deterioration is estimated to be about 15–17% in year loss for VF [24,25] and about 3–5% annual loss for VA and CS (unpublished data from the same patient sample [24] as the visual field data). Assuming a 5% annual rate of increase in logMAR as the natural course of disease, treatment with lutein resulted in significant improvement in VA at normal illumination (p value: 0.022), in low illumination VA (p value: 0.001) and also in VA at very low illumination level (p value <0.001). Also assuming a 5% annual rate of decrease in log CS, the CS at normal illumination level increased significantly (p value: 0.030), but the low (4%) and very low (0.1%) level log CS values did not show significant changes in these models. In the model assuming a probable 15–17% annual reduction in log retinal area, the changes in log retinal area during the study did not have a statistically significant difference with the expected reduction.

### Adverse effects

No significant adverse effects were seen in participants while they were taking lutein supplementation. One participant on lutein, and two participants on placebo (and multivitamin) had impaired liver function tests at one of their 6-week visits, but in all three the serum liver enzymes levels returned to the normal range when tests were repeated. Two participants had significant intolerance to multivitamin ("stomach upset") at the start f the study, prior to ingestion of lutein or placebo, and ceased multivitamin ingestion early in the study.

### Summary of results

No significant adverse effects were seen in participants while they were taking lutein supplementation. For VA at normal illumination level, treatment with lutein reduced logMAR, i.e. improved VA, but this effect was not statistically significant. The changes in normal (100%), low (4%), and very low (0.1%) illumination log CS were not statistically significant (p-values: 0.34, 0.23, and 0.32, respectively). Lutein had a statistically significant effect on VF (p-value: 0.038) and this effect increased in the model assuming a 6-week delay in effect of lutein.

## Discussion

Previous studies have suggested a beneficial effect of lutein on macular pigment [8-10,26]. As mentioned above, we are unaware of any previous randomized placebo-controlled clinical trial of lutein in RP patients. Therefore, one issue was the safety of lutein supplementation in these RP patients. Our study confirmed that lutein supplementation at 10–30 mg/day for up to 6 months is safe, with fewer adverse effects than multivitamin.

In order to have a better understanding of the effects of lutein and the pattern of these effects across time, visual function tests were collected weekly, using PC-based tests. Having this amount of data, we performed various types of analysis to test different hypotheses, although the sample size of the study to some extent restricted the strength of these further analyses. While we tested numerous models, we resolved to report the most conservative and robust estimates to make sure that we would not make unwarranted claims. Our results were consistent with what we found from analysis of visual function tests performed in the lab [19].

There is weak evidence in favor of a positive effect of lutein supplementation in preserving VA in patients with RP. As explained above, it slightly improved VA at normal illumination level, although this was not statistically significant. The non-significant results may be due to lack of enough power to detect such an effect and/or confounding by delayed effect of lutein. Considering a 6-week delay in effect of lutein, the effect size increased and even in low illumination level the non-significant negative effect of lutein changed to a positive effect. It should be underlined that there are modest improvements in VA in comparison with the natural course of the disease, which is a gradual deterioration of VA.

The evidence for the positive effect of lutein on CS was weaker, although lutein supplementation resulted in non-significant improvement of contrast sensitivity at normal and low illumination levels. Again, when compared to an expected decline due the natural course of the disease, a marginally significant benefit of lutein would be found. The log visual field area, finally, did show a beneficial effect of lutein supplementation, although the gradual loss of visual field was not significantly slowed.

One important caveat in evaluating the effects of lutein supplementation is related to the pharmacokinetics and pharmacodynamics of the supplement. Our hypothesis is that the effect of lutein starts several weeks after the start of supplementation and persists until at least several weeks after stopping the treatment. In our study we tested a six-week delay in the effect of lutein and we found several indications in support of this hypothesis. Assuming that the effects of lutein appear after 6 weeks increased the coefficients of lutein effect in VA models, i.e. increased the difference between mean logMAR of the participants when they were receiving lutein vs. when they were on placebo. Particularly, at very low illumination, the significant period effect in the original model was removed in the delayed effect model. This suggests that the period effect can be due to this delayed effect of lutein. Furthermore, stratified analysis showed that in the first half of the study lutein had a non-significant positive effect, while in the second half the relative effect of lutein was significantly negative, i.e., those no longer on 30 mg/day lutein fared better than those just starting the 10 mg/day regimen. This finding supports our hypothesis that the effect of lutein appears with some delay and may last for weeks after treatment. In other words, the significantly better VA in participants receiving placebo in the second half of the study relative to their counterparts on 10 mg/day can be the effect of high-dose lutein supplementation received in the last weeks of the first half, rather than a negative effect of low-dose lutein received by the other subject group in the second half. On the other hand, for participants who were receiving lutein in second half of the study (buildup group), there may not have been enough time for the effect of lutein to reach its optimal effective dosage, since its delayed effect appears near the end of study. Analysis of VF data under a 6-week delay assumption increased both the magnitude and the statistical significance of the effect of lutein on log retinal area. Also, our comparison of the changes in the participants' visual function with the gradual deterioration of visual function that would be expected based on previous studies, [27,28] suggests a benefit of lutein supplementation in preserving visual function.

Our results support a positive difference in the VF of RP patients taking Vitamin A, but no similar difference in VA and CS. However, in view of the relatively small number of participants using vitamin A supplementation such a difference may have been accidental.

Another caveat in our study pertains to the use of multivitamin in both groups. Some findings that could not be credited to lutein in the treatment period of our study might be attributable to one or more than one ingredients of multivitamin. In fact, in our analysis, the visual function measures in our participants while on placebo were better than the expected natural course of RP. Thus, while part of this effect might be due to the delayed effect of lutein; another explanation might be a beneficial effect of multivitamin, the only treatment during that period. Due to the small sample size of our study it is difficult to judge the generalizability of these results, yet we believe that they may apply to most patients with retinitis pigmentosa. The eligibility criteria of our study population and the method of sampling were designed to minimize the selectivity of our participants and the demographic characteristics of our study population confirmed this.

### Limitation

Three important limitations of our study were the small sample size, (justifiable for a phase I/II clinical trial), the short follow-up duration, and the lack of a wash-out period (based on the very long persistence of lutein in the eye, in macular pigment and adipose tissue) [11,16,29]. As mentioned above, the study may not have had enough power to detect minor differences between the groups; also, it was difficult to check the effect of intervention with delays of more than 6 weeks, as this would further have limited the duration of available data due to the relatively short study duration. The results seem to justify further studies of possible benefits of lutein and multivitamin use for RP patients.

## Conclusion

Lutein appears to affect visual function in ways that may differ for different measures. It has a positive effect in preserving the VF of the participants and this effect probably emerges after a number of weeks. For VA, there is modest evidence of delayed effectiveness of lutein. Finally, we did not find any significant effect of lutein in preserving CS even under the assumption of a delayed effect. Further studies with larger sample sizes and longer follow-up times will be necessary to test these hypotheses. In addition, possible effects of multivitamin on RP deserve further study. Our results suggest that a good nutritional supplement in RP may be a combination of lutein, vitamin A and multivitamin.

## Competing interests

The author(s) declare that they have no competing interests.

## Authors' contributions

HB had performed data management and statistical analysis and wrote the manuscript. MM participated in the design of the study and helped with statistical analysis. GD designed the study, supervised data collection, helped with data analysis, and participated in drafting the manuscript. All authors read and approved the final manuscript.

## Pre-publication history

The pre-publication history for this paper can be accessed here:


